# An integrative microenvironment approach for follicular lymphoma: roles of inflammatory cell subsets and immune-response polymorphisms on disease clinical course

**DOI:** 10.18632/oncotarget.27698

**Published:** 2020-08-18

**Authors:** Guilherme Rossi Assis-Mendonça, André Fattori, Rafael Malagoli Rocha, Gustavo Jacob Lourenço, Márcia Torresan Delamain, Suely Nonogaki, Vladmir Cláudio Cordeiro de Lima, Gisele Wally Braga Colleoni, Cármino Antonio de Souza, Fernando Augusto Soares, Carmen Silvia Passos Lima, José Vassallo

**Affiliations:** ^1^Department of Pathology, Faculty of Medical Sciences, University of Campinas, Campinas, São Paulo, Brazil; ^2^Laboratory of Investigative and Molecular Pathology, Faculty of Medical Sciences, University of Campinas, Campinas, São Paulo, Brazil; ^3^Department of Internal Medicine, Faculty of Medical Sciences, University of Campinas, Campinas, São Paulo, Brazil; ^4^Molecular Gynecology Laboratory, Department of Gynecology, Federal University of São Paulo, São Paulo, Brazil; ^5^Laboratory of Cancer Genetics, Faculty of Medical Sciences, University of Campinas, Campinas, São Paulo, Brazil; ^6^Hematology and Hemotherapy Center, University of Campinas, Campinas, São Paulo, Brazil; ^7^Instituto Adolfo Lutz, Secretaria de Estado da Saúde, São Paulo, Brazil; ^8^Department of Medical Oncology, AC Camargo Cancer Center, São Paulo, Brazil; ^9^Federal University of São Paulo, São Paulo, Brazil; ^10^Rede D'Or Hospitals-Pathology Department, São Paulo, Brazil

**Keywords:** follicular lymphoma, tumor microenvironment, immunohistochemistry, single nucleotide polymorphisms, prognosis

## Abstract

The study of the tumor microenvironment (TME) in follicular lymphoma (FL) has produced conflicting results due to assessment of limited TME subpopulations, and because of heterogeneous treatments among different cohorts. Also, important genetic determinants of immune response, such as single-nucleotide polymorphisms (SNPs), remain underexplored in this disease.

We performed a detailed study of the TME in 169 FL biopsies using immunohistochemistry, encompassing lymphocytes, macrophages, and cytokines. We also genotyped 16 SNPs within key immune-response genes (*IL12A, IL2, IL10, TGFB1, TGFBR1, TGFBR2, IL17A,* and *IL17F*) in 159 patients. We tested associations between SNPs, clinicopathological features and TME composition, and proposed survival models in R-CHOP/R-CVP-treated patients.

Presence of the *IL12A* rs568408 “A” allele associated with the follicular pattern of FOXP3+ cells. The *IL12A* AA haplotype included rs583911 and rs568408 and was an independent predictor of worse survival, together with the follicular patterns of T-cells (FOXP3+ and CD8+) and high IL-17F tumor levels. The patterns of CD3+, CD4+ and CD8+ cells, displayed as a principal component, also associated with survival. Hierarchical clustering of the TME proteins demonstrated a cluster that was associated with worse prognosis (tumors enriched in IL-17A, IL-17F, CD8, PD1, and Ki-67).

The survival of FL patients who were treated in the rituximab era shows a strong dependence on TME signals, especially the T-cell infiltration patterns and IL-17F tumor levels. The presence of the AA haplotype of *IL12A* in the genome of FL patients is an additional prognostic factor that may modulate the composition of T-reg cells in this disease.

## INTRODUCTION

Follicular lymphoma (FL) is the most common low-grade non-Hodgkin lymphoma (NHL) subtype and is characterized by an indolent clinical course and frequent relapses [1]. The progression of FL is influenced by stromal signals [2]. In this setting, the study of the tumor microenvironment (TME), which is composed of non-neoplastic cells and related molecules, has provided important insights on the signaling mechanisms and prognosis of FL. Several studies have described the role of tumor-infiltrating lymphocytes (TILs), macrophages, and inflammatory cytokines in the modulation of the clinical course of FL [3–9]. However, these results are often non-reproducible, due to differences in the cell quantification methods (*e.g.,* visual estimation, manual counting, automated counting) and also due to the variation in treatment patterns [10, 11].

Genes that encode inflammatory molecules, such as cytokines, also interfere in lymphoma biology, due to their intrinsic role in the development of lymphocytes [12]. In this setting, genomic variations, such as single-nucleotide polymorphisms (SNPs), especially variants that are associated with the modulation of immune functions, are relevant for the development and progression of lymphomas [12–14]. For instance, SNPs in key inflammatory genes, such as *IL10* and *IL2,* are more consistently studied in large NHL cohorts and have been implicated in disease risk or in prognosis in the pre-rituximab era, including certain FL cohorts [12, 13]. Cytokines that are encoded by other immune response genes, such as *IL12A*, *TGFB1*, *IL17A,* and *IL17F,* also regulate the balance of T-cell subsets and T-cell exhaustion in B-cell lymphomas. Nevertheless, the respective SNPs have been examined in few studies and in a non-integrated fashion with other components of the TME [14–19].

No study has yet evaluated the function of SNPs within immune genes in the TME composition of FL. This is biologically plausible, especially in the case of functional variants. In addition, there is a need for more specific prognostic factors in FL. The efficacy of rituximab (anti-CD20), which is associated with better treatment outcomes, may be influenced by components of the TME [9, 10]. Therefore a more detailed investigation of the microenvironment in patients who receive anti-CD20 is warranted.

This study aimed to verify whether SNPs in immune response genes modify the TME composition and clinical features of FL. Another goal was to test the prognostic impact of these SNPs and the TME components in a cohort of patients who have been treated with rituximab-containing regimens.

## RESULTS

### Clinicopathological features

A total of 237 FL patients were obtained (117 from UNICAMP and 120 from A. C. Camargo Cancer Center). The median age at diagnosis was of 57 years old (range: 19-94). As expected, there was a female predominance (133/237 or 56.1%), and most of the patients (189/237 or 79.7%) were diagnosed in advanced Ann Arbor stages (III or IV). The most common first-line treatment regimens employed were R-CHOP (107 patients) and R-CVP (65 patients). These and other characteristics are listed in Table 1.

**Table 1:** Clinicopathological features of the follicular lymphoma patients in this study

Characteristic	Number of patients (%)
**B symptoms**	
Present (%)	82 (34.6)
Absent (%)	150 (63.3)
Not available (%)	5 (2.1)
**FLIPI index**	
High risk (%)	73 (30.8)
Intermediate risk (%)	85 (35.9)
Low risk (%)	61 (25.7)
Not available (%)	18 (7.6)
**Bulky disease**	
Present (%)	58 (24.5)
Absent (%)	149 (62.9)
Not available (%)	30 (12.6)
**Bone marrow infiltration**	
Present (%)	102 (43.0)
Absent (%)	130 (54.8)
Not available (%)	5 (2.2)
**Extranodal disease, excluding bone marrow**	
Present (%)	77 (32.5)
Absent (%)	145 (61.2)
Not available (%)	15 (6.3)
**Ann Arbor stage**	
I or II (%)	48 (20.3)
III ou IV (%)	189 (79.7)
**Histological grade**	
1 (%)	75 (31.7)
2 (%)	92 (38.8)
3A (%)	50 (21.1)
Not available (%)	20 (8.4)
**First-line treatment**	
R-CHOP (%)	107 (45.1)
R-CVP (%)	65 (27.4)
Rituximab monotherapy (%)	4 (1.7)
CHOP/CHOP like (%)	40 (16.9)
Other drugs (%)	8 (3.4)
Watch and wait (%)	13 (5.5)

### Immunohistochemical assessment of cell components of the TME

**Figure 1:**
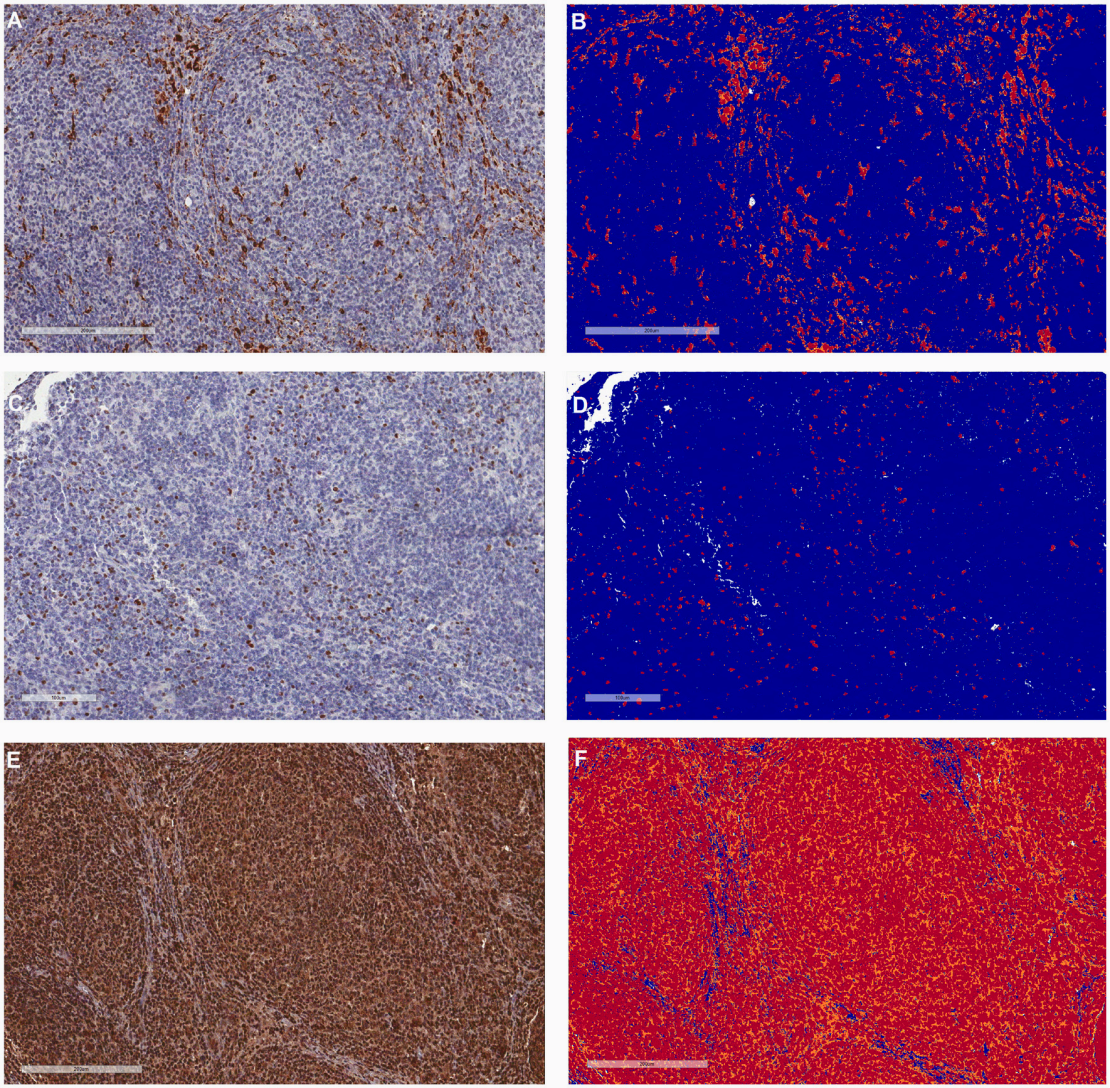
Representative pictures of the tumor microenvironment assessments by immunohistochemistry in follicular lymphoma. (**A**) and (**B**) Cytoplasmic staining of macrophages with anti-CD68 before and after pixel counting, respectively. (**C**) and (**D**) Nuclear staining of T-regulatory lymphocytes with anti-FOXP3 before and after pixel counting, respectively. (**E**) and (**F**) Diffuse cytoplasmic staining of IL-17F (high-expressor case) before and after pixel counting, respectively. The pixels were categorized as negative (blue), weak positive (yellow), positive (orange) and strong positive (red) by the Aperio system, following predetermined settings.

Immunohistochemical (IHC) analysis of the TME components was possible in samples from 169/237 patients (71.3%), which were provided as a tissue microarray (TMA). The most frequent subpopulation was T-lymphocytes (median = 21.37% of pixels), with a predominance of cells expressing CD4 (median = 18.97%) over CD8 (median = 6.09%). The least frequent subpopulation was perforin-positive cells (median = 0.28% of pixels). Regarding the expression of cytokines, IL-17A had the strongest staining score (median H-score = 209.84), and IL-12A, the weakest (median H-score = 26.47). The antibody against TGFBR2 did not pass the quality control check in the tumor samples due to non-specific staining and thus, was not further analyzed. Figure 1 illustrates representative IHC stains before and after the pixel count.

The qualitative TME assessment included an evaluation of the meshworks of follicular dendritic cells (FDCs) and the infiltration patterns of TILs. Thirty-three patients (33/147 or 22.4%) were categorized as having no detectable FDCs (Group 1). Most of the tumors (64/147 or 43.6%) were classified as having poorly developed FDC meshworks (Group 2), and 34/147 patients (23.1%) were included in Group 3, which comprised predominantly well-developed meshworks. Finally, 16/147 patients (10.9%) had uniformly well-developed meshworks (Group 4) (representative IHC images are in Supplementary Figure 1).

**Figure 2:**
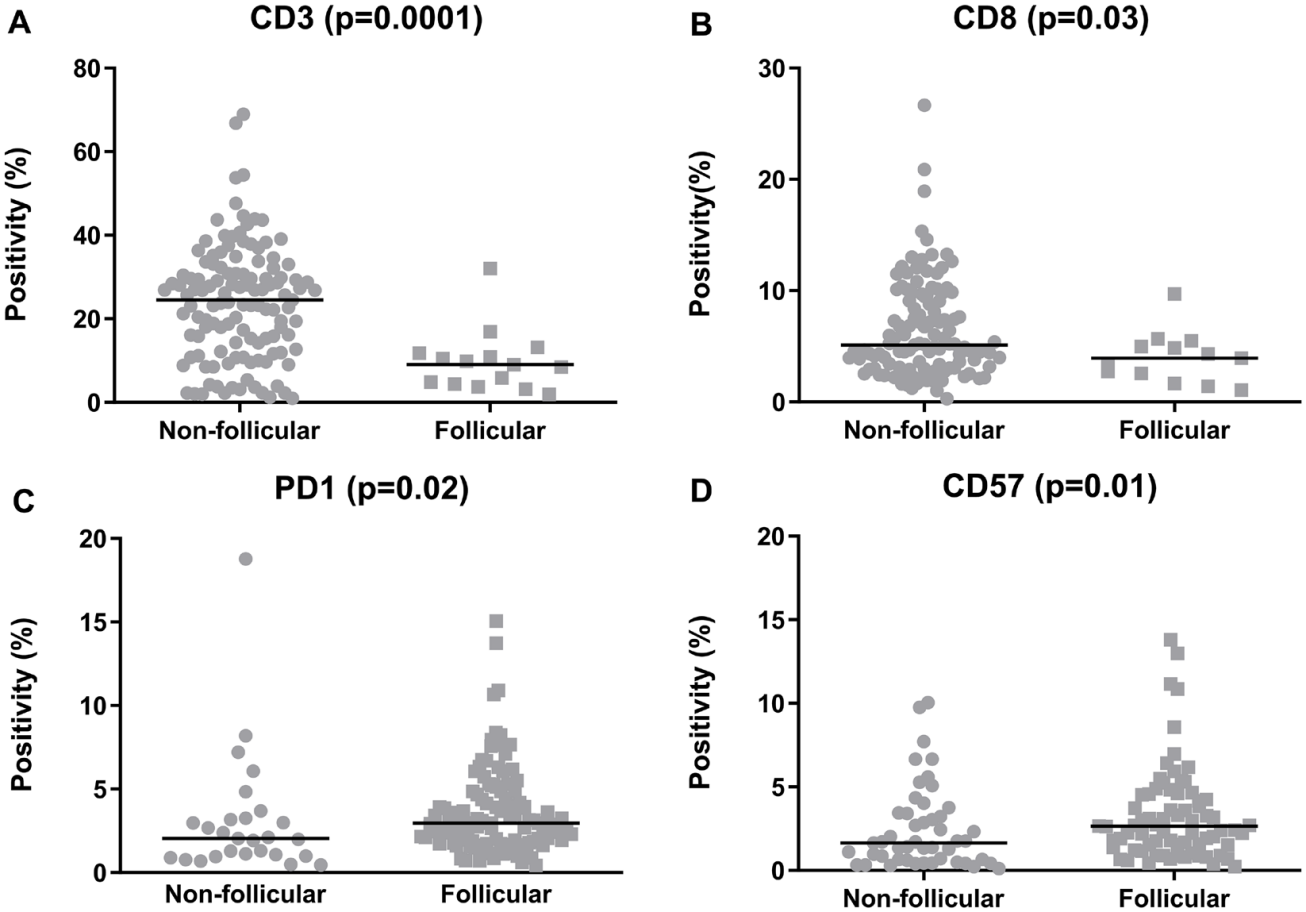
Quantification of the tumor-infiltrating lymphocytes in the follicular lymphoma samples, categorized according to the predominant infiltration pattern. (**A**) CD3, (**B**) CD8, (**C**) PD1, (**D**) CD57. The graphics show the median positivity levels and ranges, which were compared using two-tailed Mann-Whitney tests. The graphics for CD4 and FOXP3 were omitted, as no significance was achieved.

An assessment of the patterns of the TILs revealed that the CD3+, CD4+, CD8+ and FOXP3+ cells presented a non-follicular infiltration pattern in most evaluable cases (89.3%, 87.2%, 90.2% and 69.6%, respectively). In contrast, for PD1+ and CD57+ cells, the follicular pattern was more prevalent (77.9% and 60.0% of evaluable cases) (Supplementary Table 1, Supplementary Figure 2). Moreover, significant differences in cell quantities were detected between patterns. There were higher quantities of CD3+ and CD8+ cells in cases that presented with a non-follicular pattern (*p* = 0.0001 and 0.03, respectively); whereas for PD1 and CD57, there were increased cell counts in patients with a follicular pattern (*p* = 0.02 and 0.01, respectively) (Figure 2).

The presence of a follicular pattern in the CD8+ cells was associated with bone marrow infiltration at diagnosis (*p* = 0.04). However, the patterns of the other TILs did not correlate with clinical features (Supplementary Table 2).

**Figure 3:**
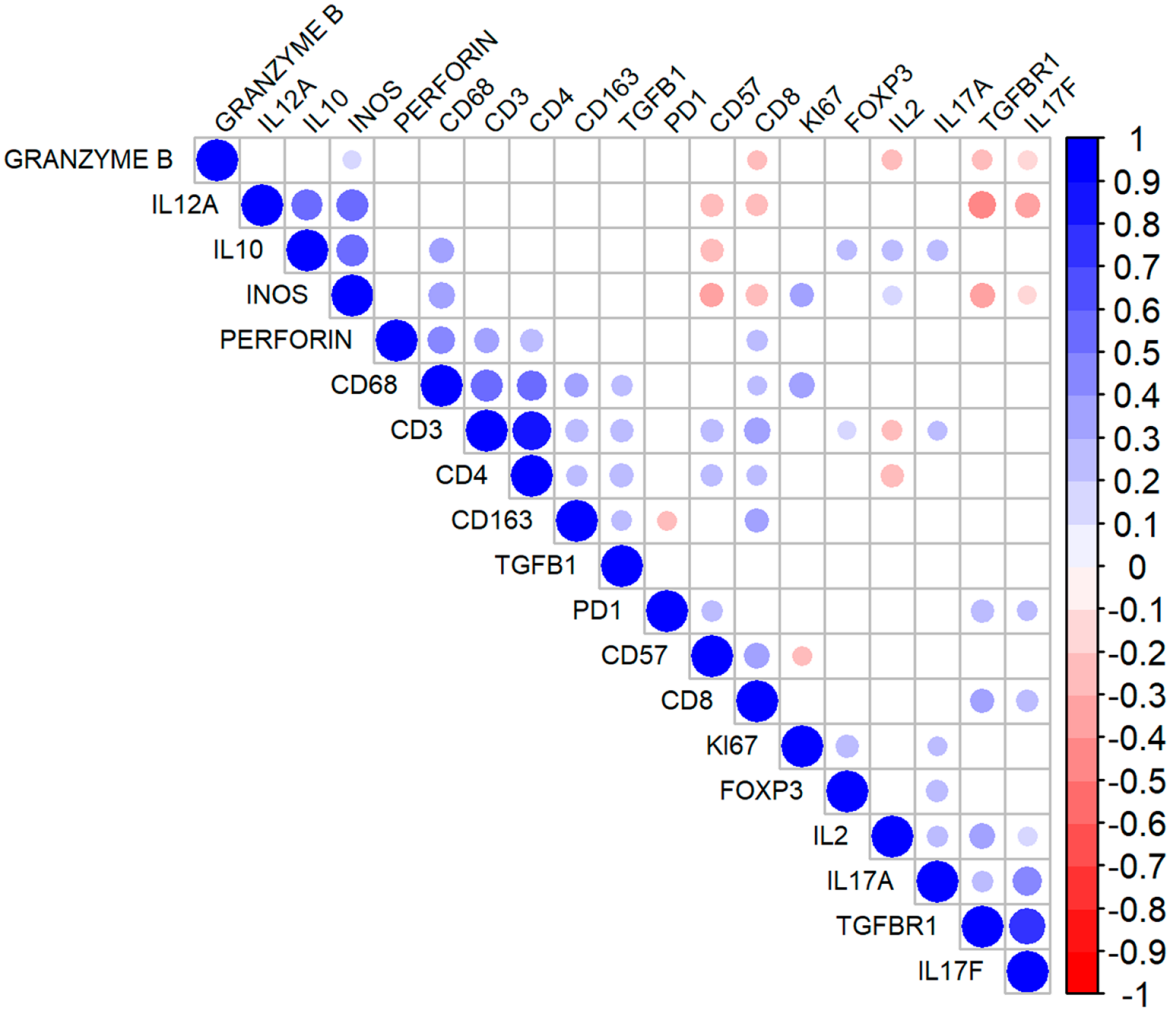
Correlation matrix for the immunohistochemical markers in follicular lymphoma. The diameter of the circles denotes higher modules of the correlation coefficient (r). The blueish tones indicate higher positive correlations, and the reddish tones denote stronger negative correlations. The non-significant correlations are indicated by the colorless intersections.

The quantifications (pixel counts) of the pan-markers (CD3 and CD68) revealed positive correlations with most other proteins. Conversely, the presence of NK cells (CD57+) correlated inversely with Ki-67, IL-12A, iNOS, and IL-10 expression. Furthermore, there was a negative correlation between the presence of CD8+ and granzyme B+ cells, indicating that not all CD8+ cells expressed cytotoxic markers. Finally, IL-17F correlated negatively with IL-12A, iNOS and granzyme B. The correlation matrix is shown in Figure 3.

The quantification of the TME cells had no impact on the clinical characteristics (data not shown). Regarding the expression of cytokines, higher IL-10 tumor levels were associated with a lower frequency of extranodal disease at diagnosis (*p* = 0.03). We also observed a trend of an association of high IL-10 levels with B-symptoms (*p* = 0.06) and with high-risk Follicular Lymphoma International Prognostic Index (FLIPI) (*p* = 0.05). The remaining cytokines were not associated with the clinical characteristics (Table 2).

**Table 2:** Intratumoral levels of cytokines and clinicopathological features of follicular lymphoma patients

	IL-12A high	IL-2 high	IL-10 high	TGFβ high	TGFBR1 high	IL-17A high	IL-17F high
**B symptoms**							
Present (%)	28/53 (52.8)	30/54 (55.5)	34/54 (62.9)	28/53 (52.8)	31/52 (59.6)	26/54 (48.1)	30/54 (55.5)
Absent (%)	55/110 (50.0)	53/109 (48.6)	49/109 (44.9)	53/106 (50.0)	48/103 (46.6)	55/108 (50.9)	52/108 (48.1)
*p*	0.97	0.53	0.06	1.00	0.48	0.73	1.00
**FLIPI**							
High risk (%)	22/45 (48.9)	21/46 (45.6)	27/46 (58.7)	21/44 (47.7)	23/42 (54.7)	26/46 (56.5)	26/46 (56.5)
Intermediate/low risk (%)	46/100 (46.0)	54/99 (54.5)	40/99 (40.4)	48/98 (49.0)	54/96 (56.2)	49/98 (50.0)	52/98 (53.0)
*p*	0.74	1.00	0.05	0.89	0.87	0.61	0.74
**Extranodal disease (excluding bone marrow)**							
Present (%)	21/50 (42.0)	22/47 (46.8)	17/49 (34.7)	22/47 (46.8)	22/46 (47.8)	27/49 (55.1)	27/48 (56.2)
Absent (%)	56/102 (54.9)	55/105 (52.3)	59/103 (57.3)	53/101 (52.4)	53/98 (54.0)	48/102 (47.0)	50/103 (48.5)
*p*	0.52	0.52	**0.03**	1.00	0.96	1.00	0.37
**Bone marrow infiltration**							
Present (%)	34/76 (44.7)	35/75 (46.7)	36/75 (48.0)	36/74 (48.6)	38/74 (51.3)	41/76 (53.9)	38/76 (50.0)
Absent (%)	48/87 (55.1)	48/88 (54.5)	46/88 (52.2)	43/85 (50.6)	39/81 (48.1)	41/86 (47.6)	43/86 (50.0)
*p*	0.36	0.62	0.58	1.00	0.91	0.84	1.00

All *p*-values were obtained by chi-squared test and were adjusted for multiple comparisons (Benjamini-Hochberg method). A “high” classification refers to the values above the median.

### Hierarchical clustering and principal component analysis of the TME components

**Figure 4:**
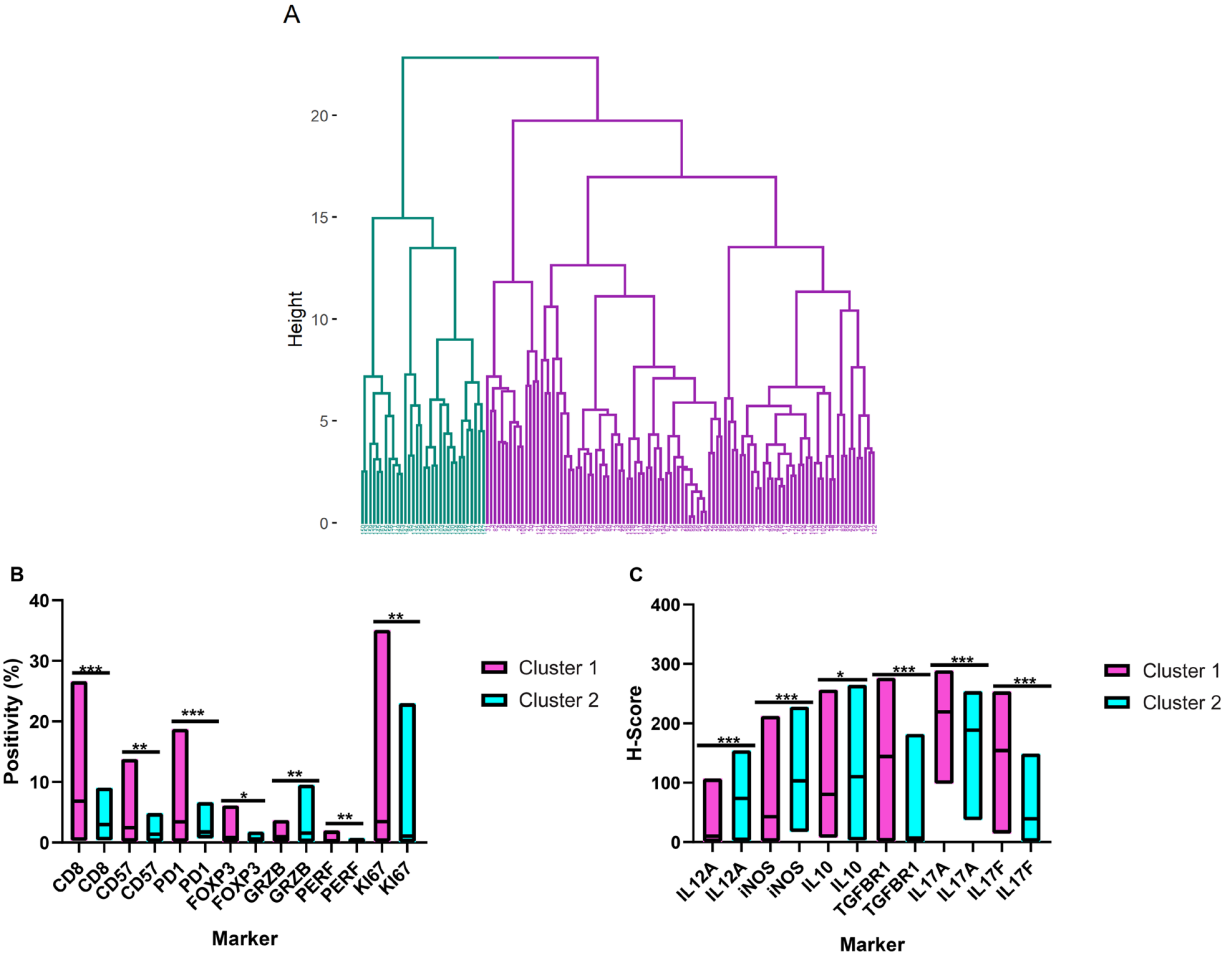
Hierarchical clustering of the tumor microenvironment in follicular lymphoma. (**A**) Dendrogram illustrating the cluster analysis of the follicular lymphoma patients based on the immunohistochemical quantifications (cluster 1 in purple and cluster 2 in blue). (**B**) and (**C**) Differential expression patterns of the tumor microenvironment cells and cytokines between clusters 1 and 2. The bars show the range of the measurements, and the horizontal lines denote the median values. ^*^ *p* ≤ 0.05; ^**^ *p* ≤ 0.01; ^***^ *p* ≤ 0.001 (two-tailed Mann-Whitney test). GRZB = Granzyme B; PERF = Perforin.

To evaluate the TME proteins using a more realistic approach, we performed unsupervised hierarchical clustering of the immunohistochemical markers that were quantified using the Aperio system. Two clusters (1 and 2) resulted, with 100 and 32 patients, respectively (Figure 4A). Cluster 1 was enriched in cells that expressed CD8, perforin, CD57, PD1, and FOXP3 and exhibited higher Ki-67 counts than cluster 2. There was also higher expression of IL-17A, IL-17F and TGFBR1 for cluster 1. In contrast, cluster 2 had higher levels of granzyme B, IL-10, IL-12A, and iNOS (Figure 4B and 4C). No differences in macrophage infiltration (CD68, CD163) were seen (data not shown).

The clinical features of clusters 1 and 2 were similar with respect to the FLIPI index, B-symptoms, and bone marrow infiltration rates. The distributions of gender and age were similar as well. However, cluster 1 had a higher frequency of extranodal disease (*p* = 0.02) (Table 3).

**Table 3:** Demographic and clinical features of follicular lymphoma patients stratified by the microenvironment clusters

	Cluster 1	Cluster 2	*p*
**Age (years)**			
≥ 57 (%)	54 (54.0)	15 (46.9)	0.54^*^
< 57 (%)	46 (46.0)	17 (53.1)	
**Gender**			
Male (%)	42 (42.0)	13 (40.7)	0.89^*^
Female (%)	58 (58.0)	19 (59.3)	
**FLIPI**			
High-risk (%)	27 (28.4)	9 (45.0)	0.14^*^
Low/intermediate risk (%)	68 (71.6)	11 (55.0)	
**B symptoms**			
Present (%)	31 (31.6)	10 (31.2)	0.96^*^
Absent (%)	67 (68.4)	22 (68.8)	
**Extranodal disease (excluding bone marrow)**			
Present (%)	36 (37.5)	4 (14.2)	0.02^**^
Absent (%)	60 (62.5)	24 (85.8)	
**Bone marrow infiltration**			
Present (%)	47 (47.0)	11 (35.5)	0.25^*^
Absent (%)	53 (53.0)	20 (64.5)	

The *p* values were obtained using Chi-squared tests (^*^) and Fisher’s exact test (^**^).

Additionally, we performed an exploratory principal component analysis (PCA) using the IHC variables (both categorical and numerical data) to complement the strictly numerical data in the clusters. The final PCA model comprised 73.01% of the variance and was composed of 6 components (Table 4). The first component showed that elevated expression of TGFBR1, IL-12A, IL-2, and IL-10 was associated with a follicular pattern in FOXP3+ cells. The second component revealed a direct association between macrophages (CD68, CD163) and perforin expression. The third component aggregated the non-follicular proliferation patterns of CD3, CD4, and CD8 cells. The fourth component revealed an association of the follicular pattern of CD8 cells with elevated intratumoral IL-10, IL-17A, and IL-17F levels. For the fifth component, high CD68 and iNOS levels were associated with a high proliferative index (Ki-67). Finally, the sixth component demonstrated an opposing trend of high TGFβ and low IL-17A levels.

**Table 4:** Principal component analysis of the tumor microenvironment in follicular lymphoma

	Component number					
	1	2	3	4	5	6
**TGFBR1**	*0.769*					
**KI67**					*0.87*	
**CD163**		*0.843*				
**IL-12A**	*0.808*					
**Perforin**		*0.793*				
**IL-10**	*0.623*			*0.572*		
**TGFβ**						*0.862*
**INOS**					*0.735*	
**CD68**		*0.629*			*0.520*	
**Pattern- CD4**			*0.832*			
**Pattern- CD8**			*0.625*	**–0.416**		
**Pattern- CD3**			*0.868*			
**Pattern- CD57**						
**Pattern- FOXP3**	**–0.460**					
**IL-17F**				*0.811*		
**IL-17A**				*0.674*		**–0.576**
**IL-2**	*0.871*					
**Individual variance**	17.65%	12.31%	12.01%	11.38%	10.84%	8.82%

The Italic and Bold text indicate the positive and negative values, respectively (range between –1 and +1). Within a single component, similar values point towards the directly associated measurements, and opposite values denote inverse associations. Considering the cell infiltration patterns, the positive values favor non-follicular patterns, and the negative numbers indicate follicular patterns.

### SNP genotyping and associations with clinicopathological features

A total of 159 FL patients (71 men and 88 women) were genotyped. There was a lack of Hardy-Weinberg Equilibrium (HWE) in 4 SNPs: *IL12A* rs568408, *IL2* rs2069762, *IL10* rs3024491, and *TGFBR1* rs334348 (Supplementary Table 3).

Some SNPs were predictors of the clinical features at diagnosis. The genotypes GT+TT of *IL2* rs6822844, compared with the GG genotype, were predictors for bulky disease at presentation (odds ratio [OR] = 2.55, 95% confidence interval [95% CI] = 1.10 – 5.91, *p* = 0.029). In addition, the presence of the CC genotype for *IL10* rs1800872, in comparison with the CA+AA genotypes, was associated with bone marrow infiltration (OR = 2.07, 95% CI: 1.07–4.01, *p* = 0.030). Regarding *IL10* rs1800890, the genotypes TA+AA were associated with the presence of B-symptoms when compared with the TT genotype (OR = 2.58, 95% CI = 1.29–5.15, *p* = 0.007). Finally, the AA genotype of *TGFB1* rs6957, compared with the AG+GG genotypes, predicted the presence of extranodal disease (OR = 2.17, 95% CI = 1.02–4.58, *p* = 0.042).

Some of the SNPs were associated with the infiltration patterns of TILs in the FL biopsies (Table 5). For instance, the follicular pattern of the FOXP3+ cells could be predicted by the GA+AA genotypes of *IL12A* rs568408 (OR = 3.07, 95% CI = 1.10–8.56, *p* = 0.03). In addition, the SNP *IL10* rs1800872 was associated with the follicular patterns of CD8 (CA+AA vs CC, OR = 8.87, 95% CI = 1.03–76.10, *p* = 0.04), PD1 (CA+AA vs CC, OR = 3.59, 95% CI = 1.17–11.0, *p* = 0.02) and CD57 (AA vs CC, OR = 8.30, 95% CI = 1.06–64.80, *p* = 0.04).

**Table 5:** Logistic regression analyses for SNPs as predictors of infiltration patterns of CD8+, PD1+, FOXP3+ and CD57+ lymphocytes

Gene and polymorphism	CD8- follicular		PD1- follicular		FOXP3- follicular		CD57- follicular	
	OR (95% CI)	*p*	OR (95% CI)	*p*	OR (95% CI)	*p*	OR (95% CI)	*p*
***IL12A*** **rs568408**								
GG	1.00 (reference)		1.00 (reference)		1.00 (reference)		1.00 (reference)	
GA+AA	3.03 (0.67–13.55)	0.14	0.73 (0.24–2.24)	0.59	3.07 (1.10–8.56)	**0.03**	2.66 (0.68–10.39)	0.15
AA	5.05 (0.39–64.73)	0.21	5^8^ (0–∞)	0.99	7.52 (0.69–81.54)	0.09	1.51 (0.14–15.75)	0.72
GG+GA	1.00 (reference)		1.00 (reference)		1.00 (reference)		1.00 (reference)	
AA	3.40 (0.30–38.73)	0.32	5^8^ (0–∞)	0.99	5.88 (0.56–61.65)	0.13	3.48 (0.34–35.23)	0.29
***IL12A*** **rs755004**								
GG	1.00 (reference)		1.00 (reference)		1.00 (reference)		1.00 (reference)	
GA+AA	1.61 (0.17–14.66)	0.67	1.18 (0.36–3.90)	0.77	1.03 (0.33–3.18)	0.95	0.78 (0.21–2.85)	0.71
***IL12A*** **rs485497**								
AA	1.00 (reference)		1.00 (reference)		1.00 (reference)		1.00 (reference)	
AG+GG	0.75 (0.13–4.12)	0.74	1.34 (0.45–3.98)	0.59	0.43 (0.15–1.22)	0.11	0.19 (0.06–0.58)	**0.003**
GG	0.74 (0.09–6.03)	0.78	1.47 (0.75–2.88)	0.25	0.56 (0.15–2.12)	0.39	0.30 (0.07–1.24)	0.09
AA+AG	1.00 (reference)		1.00 (reference)		1.00 (reference)		1.00 (reference)	
GG	0.94 (0.17–5.14)	0.94	2.53 (0.85–7.48)	0.09	1.00 (0.34–2.90)	0.99	0.70 (0.19–2.47)	0.58
***IL12A*** **rs583911**								
AA	1.00 (reference)		1.00 (reference)		1.00 (reference)		1.00 (reference)	
AG+GG	2.56 (0.28–22.70)	0.39	3.46 (1.20–9.97)	**0.02**	2.07 (0.71–6.01)	0.17	1.88 (0.60–5.88)	0.27
GG	0.41 (0.03–5.18)	0.49	0.29 (0.06–1.27)	0.10	0.74 (0.17–3.11)	0.68	1.17 (0.30–4.53)	0.81
AA+AG	1.00 (reference)		1.00 (reference)		1.00 (reference)		1.00 (reference)	
GG	1.43 (0.24–8.31)	0.68	2.01 (0.51–7.80)	0.31	0.88 (0.29–2.61)	0.82	0.40 (0.13–1.27)	0.12
***IL2* rs2069762 **								
TT	1.00 (reference)		1.00 (reference)		1.00 (reference)		1.00 (reference)	
TG+GG	1.45 (0.30–7.06)	0.63	0.58 (0.21–1.60)	0.30	1.35 (0.54–3.32)	0.51	0.65 (0.24–1.80)	0.41
***IL2* rs6822844 **								
GG	1.00 (reference)		1.00 (reference)		1.00 (reference)		1.00 (reference)	
GT+TT	N/E	N/E	2.67 (0.55–12.81)	0.21	0.78 (0.25–2.37)	0.66	0.56 (0.18–1.75)	0.32
***IL10* rs1800872 **								
CC	1.00 (reference)		1.00 (reference)		1.00 (reference)		1.00 (reference)	
CA+AA	8.87 (1.03–76.10)	**0.04**	3.59 (1.17–11.0)	**0.02**	2.45 (0.99–6.06)	0.05	2.43 (0.87–6.77)	0.08
AA	N/E	N/E	6.75 (1.27–35.89)	**0.02**	1.58 (0.32–7.63)	0.56	8.30 (1.06–64.80)	**0.04**
CC+CA	1.00 (reference)		1.00 (reference)		1.00 (reference)		1.00 (reference)	
AA	N/E	N/E	3.44 (0.80–14.69)	0.09	1.02 (0.22–4.58)	0.97	5.35 (0.84–33.94)	0.07
***IL10* rs3024491 **								
CC	1.00 (reference)		1.00 (reference)		1.00 (reference)		1.00 (reference)	
CA+AA	5.17 (0.57–47.00)	0.14	2.93 (0.78–10.88)	0.10	1.03 (0.35–2.97)	0.95	1.22 (0.39–3.76)	0.72
AA	N/E	N/E	N/E	N/E	0.63 (0.05–7.06)	0.71	1.84 (0.15–21.83)	0.62
CC+CA	1.00 (reference)		1.00 (reference)		1.00 (reference)		1.00 (reference)	
AA	N/E	N/E	N/E	N/E	0.56 (0.05–5.93)	0.63	1.40 (0.13–14.79)	0.77
***IL10* rs1800890 **								
TT	1.00 (reference)		1.00 (reference)		1.00 (reference)		1.00 (reference)	
TA+AA	4.25 (0.72–24.91)	0.10	2.13 (0.52–8.71)	0.29	2.44 (0.83–7.16)	0.10	1.52 (0.47–4.88)	0.48
AA	N/E	N/E	N/E	N/E	1.29 (0.10–16.01)	0.84	N/E	N/E
TT+TA	1.00 (reference)		1.00 (reference)		1.00 (reference)		1.00 (reference)	
AA	N/E	N/E	N/E	N/E	0.87 (0.07–10.46)	0.91	N/E	N/E
***TGFB1* rs1800469 **								
CC	1.00 (reference)		1.00 (reference)		1.00 (reference)		1.00 (reference)	
CT+TT	2.17 (0.37–12.61)	0.38	1.39 (0.41–4.69)	0.59	0.81 (0.27–2.36)	0.70	0.72 (0.23–2.23)	0.57
TT	22.8 (1.15–450.48)	**0.04**	0.93 (0.07–11.22)	0.95	1.78 (0.20–15.43)	0.60	0.57 (0.02–11.90)	0.72
CC+CT	1.00 (reference)		1.00 (reference)		1.00 (reference)		1.00 (reference)	
TT	24.38 (1.62–366.71)	**0.02**	0.64 (0.06–6.77)	0.71	1.95 (0.25–15.27)	0.52	0.51 (0.02–9.19)	0.65
***TGFB1* rs1800471 **								
GG	1.00 (reference)		1.00 (reference)		1.00 (reference)		1.00 (reference)	
GC+CC	1.92 (0.19–19.28)	0.57	N/E	N/E	2.22 (0.49–10.03)	0.29	1.21 (0.21–6.90)	0.82
***TGFB1* rs6957 **								
AA	1.00 (reference)		1.00 (reference)		1.00 (reference)		1.00 (reference)	
AG+GG	2.18 (0.45–10.47)	0.33	1.04 (0.38–2.87)	0.93	1.50 (0.61–3.66)	0.37	1.09 (0.39–3.00)	0.86
GG	N/E	N/E	0.10 (0.00–1.35)	0.08	N/E	N/E	N/E	N/E
AA+AG	1.00 (reference)		1.00 (reference)		1.00 (reference)		1.00 (reference)	
GG	N/E	N/E	0.07 (0.00–1.00)	0.05	N/E	N/E	N/E	N/E
***TGFBR1* rs334348 **								
AA	1.00 (reference)		1.00 (reference)		1.00 (reference)		1.00 (reference)	
AG+GG	1.73 (0.31–9.72)	0.52	2.67 (0.96–7.43)	0.05	0.83 (0.33–2.09)	0.69	0.87 (0.31–2.43)	0.79
GG	N/E	N/E	N/E	N/E	N/E	N/E	0.54 (0.02–12.90)	0.70
AA+AG	1.00 (reference)		1.00 (reference)		1.00 (reference)		1.00 (reference)	
GG	N/E	N/E	N/E	N/E	N/E	N/E	0.67 (0.03–12.28)	0.79
***TGFBR2* rs3087465 **								
GG	1.00 (reference)		1.00 (reference)		1.00 (reference)		1.00 (reference)	
GA+AA	0.52 (0.11–2.40)	0.40	0.73 (0.27–1.95)	0.54	1.39 (0.57–3.37)	0.46	0.94 (0.35–2.56)	0.91
AA	N/E	N/E	0.07 (0.0–0.84)	**0.03**	2.58 (0.30–22.13)	0.38	N/E	N/E
GG+GA	1.00 (reference)		1.00 (reference)		1.00 (reference)		1.00 (reference)	
AA	N/E	N/E	0.07 (0.0–0.76)	**0.03**	2.17 (0.27–17.19)	0.46	N/E	N/E
***IL17A* rs3748067 **								
CC	1.00 (reference)		1.00 (reference)		1.00 (reference)		1.00 (reference)	
CT+TT	0.31 (0.03–2.81)	0.30	0.50 (0.18–1.41)	0.19	0.70 (0.25–1.93)	0.49	0.59 (0.20–1.70)	0.33
***IL17F* rs763780 **								
TT	1.00 (reference)		1.00 (reference)		1.00 (reference)		1.00 (reference)	
TC+CC	0.63 (0.10–3.64)	0.60	0.40 (0.14–1.15)	0.09	0.70 (0.26–1.87)	0.48	0.63 (0.20–1.92)	0.41

OR (95%CI) = Odds ratio with 95% confidence interval. N/E = Not evaluable (OR = zero or OR = ∞).

**Figure 5:**
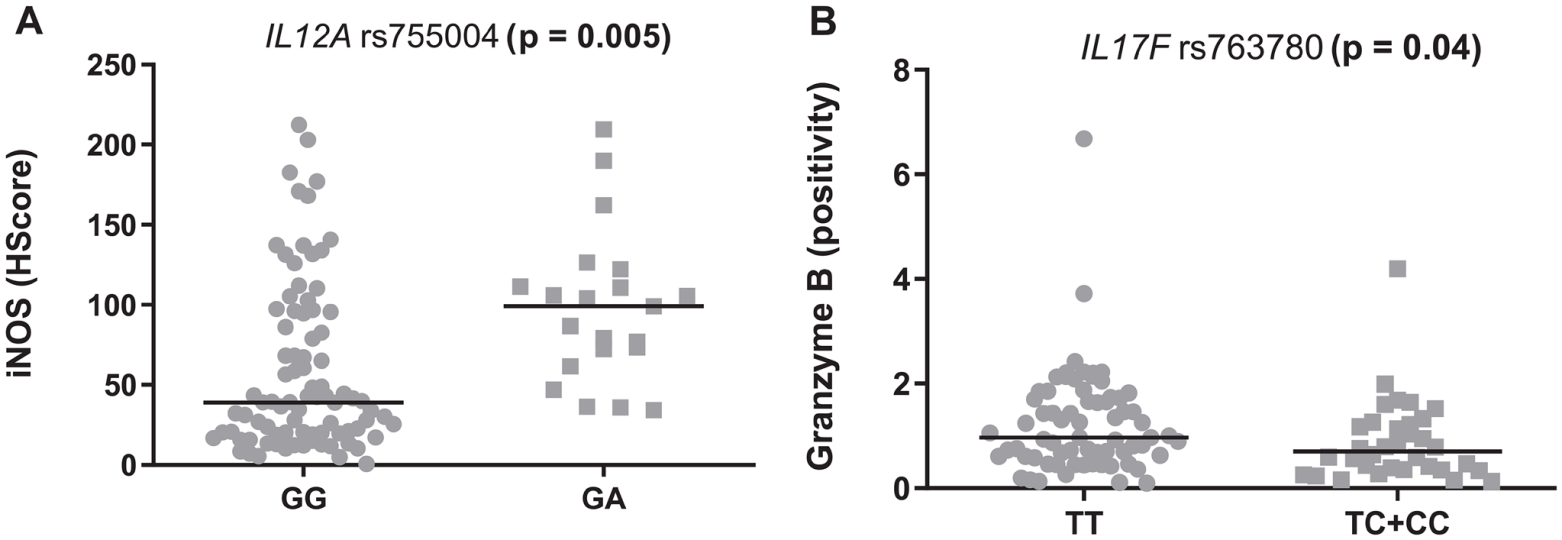
Modulation of the tumor microenvironment in follicular lymphoma by inflammatory SNPs. (**A**) rs755004 genotypes and iNOS levels. (**B**) rs763780 and granzyme B levels. The graphics show the median positivity levels and ranges, which were compared using two-tailed Mann-Whitney tests. The *p* values were adjusted for multiple comparisons (Benjamini-Hochberg method).

Other SNPs also associated with the quantification of TME proteins. First, a higher expression of iNOS was seen in the samples from FL patients carrying the “A” allele of *IL12A* rs755004 (*p* = 0.005, Figure 5A). Second, a higher percentage of granzyme B+ cells was observed in tumors from patients with the TT genotype of *IL17F* rs763780 (*p* = 0.04, Figure 5B). However, none of the SNPs altered the levels of the proteins that were encoded by each of their respective genes (data not shown).

### Survival analyses

One-hundred seventy-two patients received R-CHOP or R-CVP as a first-line treatment and therefore were included in the survival analyses. The median follow-up times were of 6.05 years (all patients) and 6.32 years (living patients). At the end of the follow-up, 136 patients were alive (79.06%) and 36 (20.94%) had died.

We first evaluated the role of the main clinical features in event-free survival (EFS) and overall survival (OS) by estimating their hazard ratios (HRs) by univariate Cox regressions. As expected, the presence of high-risk FLIPI was associated with worse EFS (HR = 1.77, 95% CI: 1.08–2.88, *p* = 0.02) and OS (HR = 3.29, 95% CI: 1.64–6.59, *p* = 0.001) by univariate analysis. The same was observed for the presence of B-symptoms (HR = 2.39, 95% CI: 1.49–3.86, *p* < 0.001 for EFS and HR = 4.00, 95% CI: 2.02–7.93, *p* < 0.001 for OS). The presence of extranodal disease (excluding bone marrow) was also associated with worse EFS (HR = 1.86, 95% CI: 1.15–3.01, *p* < 0.01) and OS (HR = 2.48, 95% CI: 1.23–4.98, *p* < 0.01). Bone marrow infiltration was marginally associated with worse OS (HR = 1.89, 95% CI: 0.96–3.74, *p* = 0.06), but not with EFS (HR = 1.39, 95% CI: 0.87–2.22, *p* = 0.15). Finally, the administration of maintenance rituximab was associated with prolonged OS (HR = 0.40, 95% CI: 0.19–0.85, *p* = 0.01), but did not affect EFS (HR = 0.67, 95% CI: 0.41–1.09).

**Figure 6:**
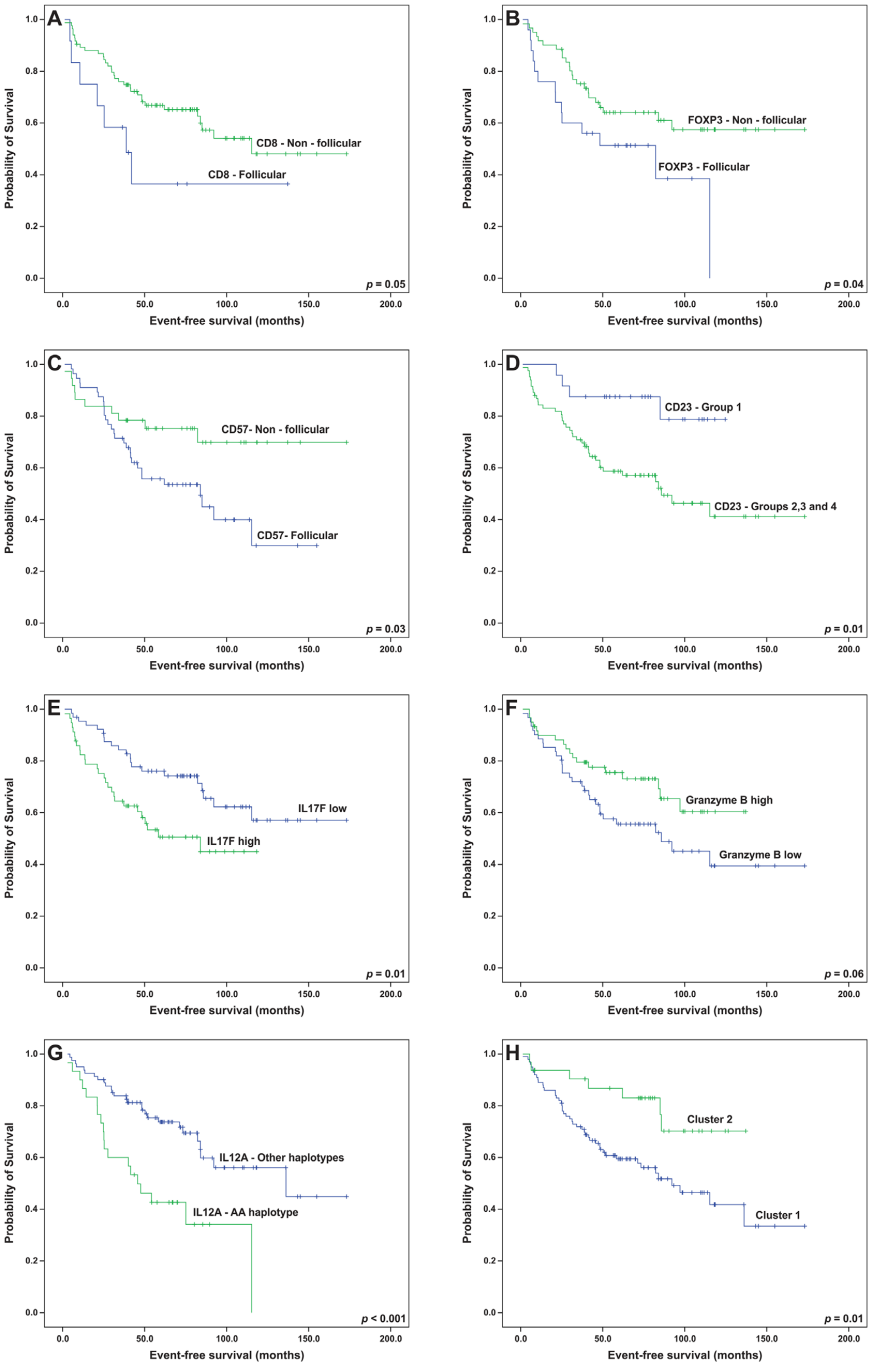
Event-free survival analyses (Kaplan-Meier curves) of follicular lymphoma patients treated with R-CHOP/R-CVP influenced by the patterns of (**A**) CD8, (**B**) FOXP3, (**C**) CD57 and (**D**) CD23, (**E**) intratumoral levels of IL-17F, (**F**) intratumoral levels of granzyme B, (**G**) the presence of the *IL12A* AA haplotype and (**H**) microenvironment clusters. All the *p*-values were obtained from log-rank tests.

**Figure 7:**
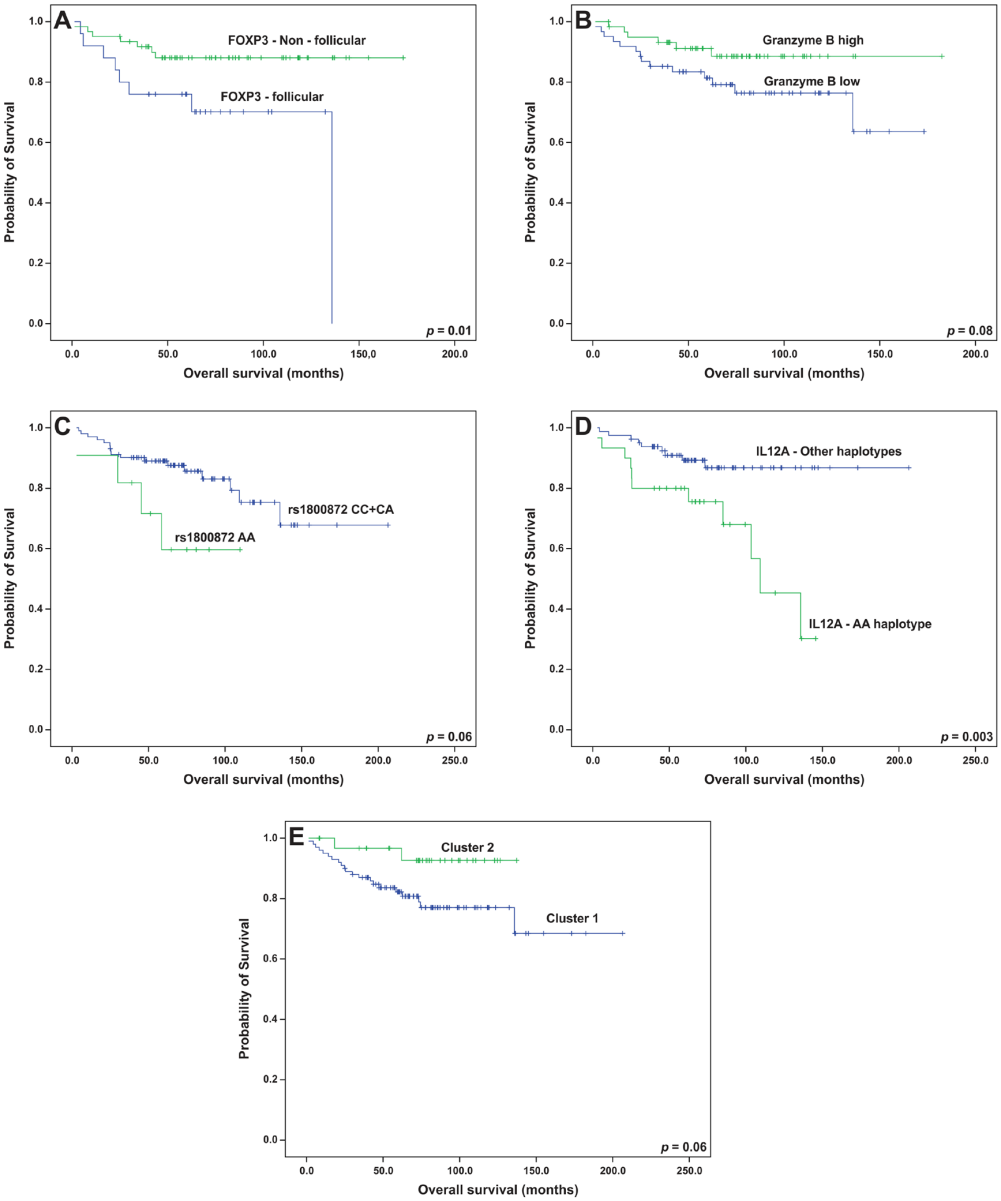
Overall survival analyses (Kaplan-Meier curves) of follicular lymphoma patients treated with R-CHOP/R-CVP influenced by (**A**) the pattern of FOXP3 cells, (**B**) intratumoral levels of granzyme B, (**C**) the genotypes of *IL10* rs1800872, (**D**) the presence of the *IL12A* AA haplotype and (**E**) microenvironment clusters. All the *p*-values were obtained from log-rank tests.

For the univariate analysis, the presence of follicular patterns for CD8 and CD57 was associated with worse EFS (*p* = 0.05 and 0.03, respectively); whereas, the follicular pattern of FOXP3 was associated with both worse EFS (*p* = 0.04) and OS (*p* = 0.01) (Kaplan-Meier curves on Figures 6A–6C and 7A). In addition, high immunohistochemical expression of IL-17F and the presence of FDC meshworks (Groups 2, 3 and 4) were associated with worse EFS (*p* = 0.01 for both markers, Kaplan-Meier curves on Figure 6D and 6E). Finally, a trend toward improved EFS (*p* = 0.06) and OS (*p* = 0.08) was seen in cases with high number of cells that expressed granzyme B (Figures 6F and 7B). After the multivariate analysis, the immunohistochemical variables that were independently associated with worse EFS included a follicular pattern of CD8+ cells (*p* = 0.001) and elevated expression of IL-17F (*p* = 0.04). Conversely, the presence of a follicular pattern of FOXP3+ cells was the only variable that was independently associated with worse OS (*p* = 0.02) (Table 6).

**Table 6:** Cox regression for survival of follicular lymphoma patients

Variable	Univariate				Multivariate*			
	EFS HR (95% CI)	*p*	OS HR (95% CI)	*p*	EFS HR (95% CI)	*p*	OS HR (95% CI)	*p*
**Immunohistochemical model**								
**Pattern – CD8**								
Follicular	2.17 (0.95–4.93)	0.06	2.35 (0.77–7.16)	0.13	10.99 (2.78–43.42)	**0.001**	N/A	N/A
Non-follicular	Reference		Reference		Reference		Reference	
**Pattern – FOXP3**								
Follicular	1.96 (1.00–3.84)	**0.04**	3.25 (1.17–9.03)	**0.02**	1.42 (0.62–3.25)	0.40	7.78 (1.40–43.22)	**0.02**
Non-follicular	Reference		Reference		Reference		Reference	
**Pattern - CD57**								
Follicular	2.18 (1.05–4.49)	**0.04**	1.04 (0.37–2.86)	0.93	1.02 (0.42–2.50)	0.95	N/A	N/A
Non-follicular	Reference		Reference		Reference		Reference	
**IL-17F**								
High	2.08 (1.16–3.71)	**0.01**	1.98 (0.82–4.78)	0.12	2.32 (1.01–5.31)	**0.04**	N/A	N/A
Low	Reference		Reference		Reference		Reference	
**Granzyme B**								
High	0.57 (0.32–1.03)	0.06	0.44 (0.17–1.15)	0.09	0.67 (0.28–1.58)	0.36	0.37 (0.07–1.88)	0.23
Low	Reference		Reference		Reference		Reference	
**CD23 group**								
1	Reference		Reference		Reference		Reference	
2, 3 or 4	3.55 (1.26–9.95)	**0.01**	31.25 (0.28–3467.8)	0.15	1.66 (0.37–7.39)	0.50	N/A	N/A
**Genetic model**								
***IL12A*** **AA haplotype** (rs583911 and rs568408)								
Present	2.80 (1.54–5.11)	**0.001**	3.49 (1.44–8.43)	**0.005**	2.25 (1.18–4.27)	**0.01**	2.82 (0.89–8.92)	0.07
Absent	Reference		Reference		Reference		Reference	
**rs1800872** (***IL10***)								
AA	1.28 (0.50–3.24)	0.59	2.75 (0.91–8.27)	0.07	N/A	N/A	1.03 (0.37–2.88)	0.96
AC+CC	Reference		Reference		Reference		Reference	
**Principal components model**								
**Third microenvironment component (numerical value)**	0.68 (0.49–0.95)	**0.02**	0.81 (0.48–1.35)	0.42	0.59 (0.40–0.89)	**0.01**	N/A	N/A

EFS = event-free survival; OS = overall survival; HR = hazard ratio; 95% CI = 95% confidence interval; FLIPI: Follicular Lymphoma International Prognostic Index; (*) Adjustment for FLIPI, B-symptoms and extranodal disease (EFS); adjustment for FLIPI, B-symptoms, extranodal disease, bone marrow infiltration and the use of rituximab as maintenance therapy (OS).

**Figure 8:**
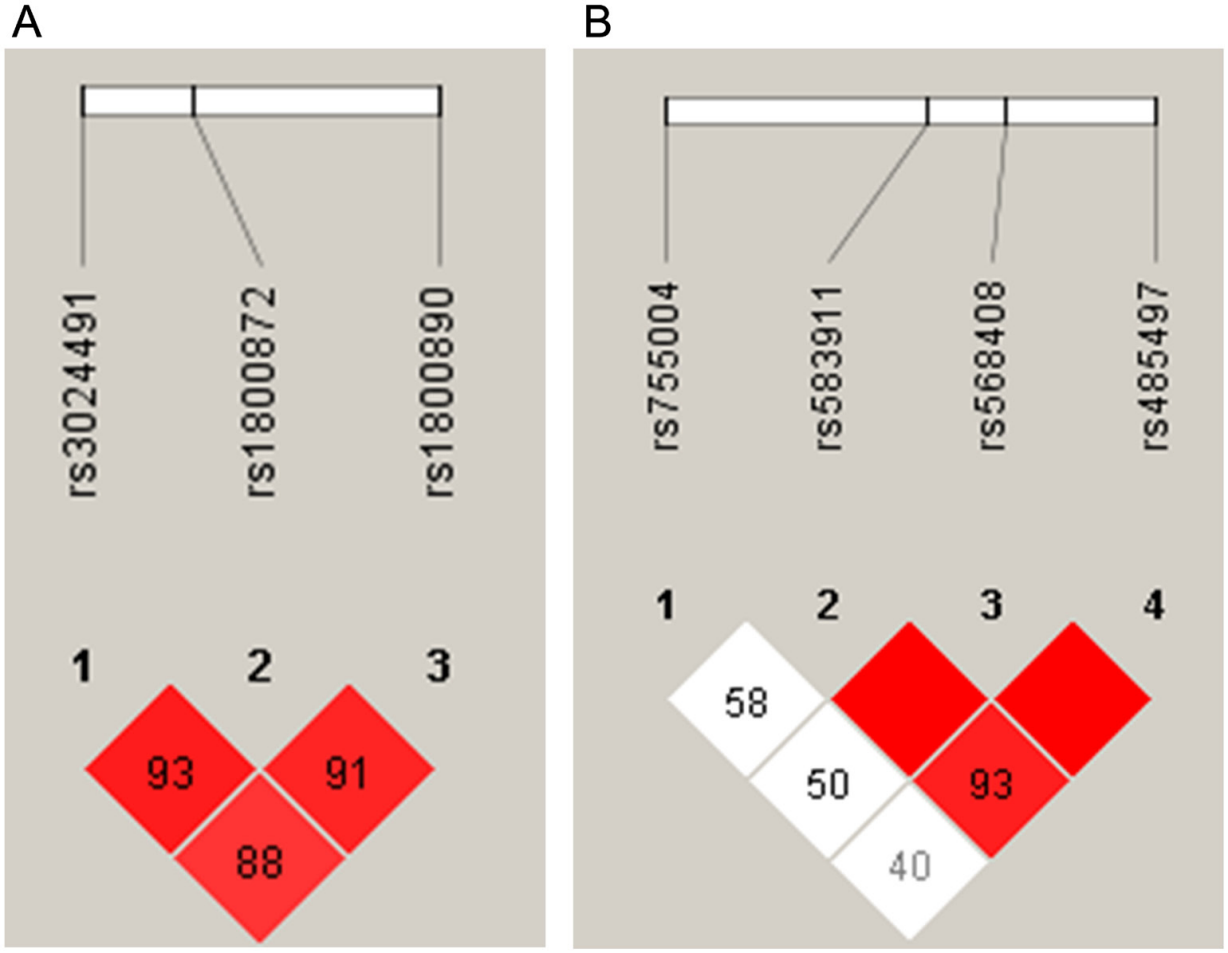
Haplotype estimates in follicular lymphoma patients for (**A**) chromosome 1 (*IL10*) and (**B**) chromosome 3 (*IL12A*). In each square, the linkage disequilibrium (LD) was estimated between the groups of single nucleotide polymorphisms. The higher LD values (expressed as D’) are shown in the red squares.

The prognostic roles of all SNPs and haplotypes were also evaluated. Linkage disequilibrium (LD) analyses allowed for the construction of haplotypes in *IL10* and *IL12A* (Figure 8; frequencies of all the haplotypes in Supplementary Table 4). The AA-genotype of the polymorphism *IL10* rs1800872 was slightly associated with worse OS by univariate analysis (*p* = 0.06, Figure 7C). In addition, the presence of a haplotype on *IL12A* (AA), involving the rs583911 and rs568408 “A” alleles, was associated with worse EFS and OS (*p* < 0.001 and *p* = 0.003, respectively; Kaplan-Meier curves in Figures 6G and 7D). After the multivariate analysis, the AA haplotype remained significant for EFS (*p* = 0.01) and was marginally associated with OS (*p* = 0.07) (Table 6).

Finally, we examined whether variables from the dimension-reducing analyses (PCA and clusters) altered the survival rates. In this setting, the presence of the third principal component was associated with prolonged EFS. In other words, patients with non-follicular infiltration patterns for CD3, CD4 and CD8 lymphocytes had better EFS (*p* = 0.02), and the statistical significance was maintained after the multivariate analysis (*p* = 0.01, Table 6). Complementarily, patients in cluster 1 had poorer EFS (*p* = 0.01) and a tendency toward worse OS (*p* = 0.06) than those in cluster 2 (Figures 6H and 7E). After adjusting for FLIPI, cluster 1 remained an independent predictor of worse EFS (*p* = 0.03) (Table 7).

**Table 7:** Univariate and FLIPI-adjusted Cox regressions for the microenvironment clusters in follicular lymphoma patients

	Univariate				FLIPI-adjusted			
	EFS HR (95% CI)	*p*	OS HR (95% CI)	*p*	EFS HR (95% CI)	*p*	OS HR (95% CI)	*p*
**Cluster**								
1	2.57 (1.16–5.70)	**0.02**	3.54 (0.82–15.15)	0.08	2.91 (1.08–7.32)	**0.03**	6.66 (0.88–50.47)	0.06
2	Reference		Reference		Reference		Reference	
**FLIPI**								
High risk	1.77 (1.08–2.88)	**0.007**	3.29 (1.64–6.59)	**0.001**	2.03 (1.12–3.68)	**0.03**	4.32 (1.80–10.36)	**0.001**
Low/intermediate risk	Reference		Reference		Reference		Reference	

EFS = event-free survival; OS = overall survival; HR = hazard ratio; 95% CI = 95% confidence interval; FLIPI: Follicular Lymphoma International Prognostic Index.

## DISCUSSION

We found that in FL patients uniformly treated with R-CHOP/R-CVP, the T-cell infiltration patterns, more importantly than quantification of these cells, independently associate with prognosis. In addition, we described a novel adverse and independent prognostic role of the AA haplotype in *IL12A*.

We reproduced previous successful evaluations of T-cell architectural patterns using TMAs [5, 7, 20] and found that the follicular pattern for FOXP3+ cells (T-reg cells) was independently associated with worse OS. Farinha et al. (2010) reported that this parameter shortened the time to transformation of FL in the pre-rituximab era [5]. We hereby confirm that this adverse impact also exists in patients receiving anti-CD20 therapy. In FL, FOXP3+ cells suppress the immune function of other T-cells, impairing immunosurveillance [21]. Also, FL cells secrete CCL22, which induces the recruitment of T-reg cells [22]. Hence, it is conceivable that the concentration of T-reg cells near the FL cells (follicular pattern) is a histological representation of these immunosuppressive interactions.

Our findings also demonstrate that the follicular pattern of CD8+ cells was an independent factor for worse EFS, which is a novel finding for FL. In addition, one principal component encompassing non-follicular proliferations of T cell subsets (CD3+, CD4+ and CD8+), showed a favorable impact on EFS, which complements what was observed inside the neoplastic follicles. Indeed, T-cells in the interfollicular compartment seem to have preserved immune synapses and enhanced granzyme B production, possibly due to the involvement of the “border patrolling” effect [7, 23, 24]. In this setting, our results are plausible and corroborate the findings of Wahlin et al. (2010), associating non-follicular CD8+ cells with a good outcome in FL patients who have been treated in the pre-rituximab era [7].

Conversely, the presence of CD8+ lymphocytes in direct contact with FL cells (*i.e.* follicular proliferation pattern) promotes a defective immunological synapse due to the impairment of the CD8+ cell’s F-actin cytoskeleton [25]. In addition, a subset of T-CD8+ cells in FL, located in the follicular compartment, may co-express molecules, such as ETV1, impairing immune function [26]. Therefore, it is plausible that CD8+ cells that are arranged in follicular patterns represent anergic subpopulations, which is reinforced by our finding associating the follicular patterns of CD8+ cells with worse outcomes.

Our initial survival analysis, as discussed above, showed that the T-cell patterns were associated with prognosis. However, none of the individual cell quantities significantly influenced survival. Notably, when we collectively evaluated these cells by hierarchical clustering, it became evident that the patients from cluster 1 had a poorer survival, independent of the FLIPI. Cluster 1 was enriched in cells with a high proliferation index (Ki-67) and in some T-cell subsets (CD8+, PD1+, FOXP3+). This cluster also presented with elevated expression of TGFBR1, IL-17A, and IL-17F. These results suggest that the quantities of the inflammatory cells, including T-cells, are important when they interact with each other and with cytokines, which reflects the complexity of TME in FL [27]. The mechanisms of these interactions should be explored further in future studies.

However, IL-17F was overexpressed in cluster 1 and was also found individually as an adverse prognostic factor for EFS. A previous report associated IL-17F expression with a bad clinical outcome of T-cell lymphomas, whereas for B-cell neoplasms, the scenario was unclear [14, 15]. In this study, we described a novel adverse prognostic role for IL-17F in FL patients, mirroring what has been observed in T-cell lymphomas. The mechanism of this association has not been described, but it may reside in the downstream effects of IL-17F, such as angiogenic sprouting and prostaglandin production [14].

To complement the TME investigation in FL, we examined the genetics of the host. The AA haplotype of *IL12A* (constituted by rs583911 and rs568408 “A” alleles) was independently associated with worse EFS and was marginally associated with OS. Two previous studies did not find any association between rs568408 and outcome in FL. However, the cohorts in these studies were predominantly from the pre-rituximab era, limiting a direct comparison with our results [13, 28]. The explanation for the prognostic role of the AA haplotype may reside in the association between the “A” allele of rs568408 and the follicular pattern of the distribution of the FOXP3+ cells. However, the exact mechanism of this association has not been elucidated. It is known that the rs568408 “A” allele may lower the activity of IL-12A by disrupting exonic splicing sites [29]. In this setting, a microenvironment with less active IL-12A may favor the outgrowth of fully immunosuppressive T-reg cells, which in FL, seems to be represented by such an arrangement as a follicular pattern, according to our results and others [5, 30].

It is possible that other SNPs modulate the TME composition without altering the course of FL. For instance, the “A” allele of *IL12A* rs755004 was associated with increased intratumoral iNOS levels, which may be explained by the role of IL-12A on IFNγ secretion, with the consequent recruitment of fagocytes and oxidative burst [31]. We also found that *IL17F* rs763780 was associated with granzyme B levels, which may relate to the interference of Th17 cells on cytotoxic lymphocytes [32].

The most significant limitation of our study was its retrospective design. Therefore, the validation of our results using prospective cohorts is warranted. However, several strengths must be recognized. For example, our study had a relatively large sample size, and the patients were homogeneously treated with rituximab for our survival analyses, minimizing any therapy-related biases. We also performed computer-assisted image analyses for the quantification of the IHC markers, which has been proven superior and more reproducible in comparison to simple manual counting [11].

In this study, we demonstrated the adverse prognostic role of several TME elements in FL, considering both protein and genetic data. Importantly, the follicular pattern of the T-CD8+ cells, the high expression of IL-17F (individually and as a cluster member), and the AA haplotype of *IL2A* were all independently associated with worse prognosis. The latter variable was also associated with the pattern of FOXP3+ cell infiltration, which was validated in this study as a prognostic factor in the rituximab era. We believe that the simultaneous study of the tumor biopsies and immune response related SNPs in patients’ peripheral blood allowed for a novel and more integrative approach that will provide new insights on follicular lymphoma biology.

## MATERIALS AND METHODS

### Patients, controls and clinical data

The present retrospective study included an initial number of 237 cases, diagnosed with systemic FL (grades 1, 2, or 3A) between 1999 and 2016 at the Hematology and Hemotherapy Center – UNICAMP and A. C. Camargo Cancer Center.

The clinical data were collected from the medical files. FLIPI was used as the reference prognostic tool.

The study was approved by the Ethics Committees of both institutions (number 32177014.3.0000.5404), and all procedures were carried out according to the Declaration of Helsinki.

### SNP selection and genotyping

The SNPs were studied using a “candidate gene” approach. SNPs located in immune response genes from the Th1, Th2, or Th17 axis, with previous evidence of active roles in lymphoma, cancer or immune response, were selected. A minor allele frequency of 5% was required. Sixteen SNPs were then selected, based on previous association studies (Table 8) [13, 18, 19, 29, 33–51].

**Table 8:** Single nucleotide polymorphisms (SNPs) assessed in this study

Gene	SNP	Previous associations^*^	References^*^
***IL12A***	rs755004	Risk for NHL	[19]
***IL12A***	rs583911	Risk for ALL	[33]
***IL12A***	rs568408	Risk for FL; alterations in *IL12A* splicing sites	[29, 34]
***IL12A***	rs485497	Risk for FL; modulation of IL-12A serum levels	[35, 36]
***IL2***	rs2069762	Risk for NHL; prognostic factor in FL patients (pre-rituximab era)	[13, 37]
***IL2***	rs6822844	Association with multiple autoimmune diseases	[38]
***IL10***	rs3024491	Modulation of IL-10 production	[39]
***IL10***	rs1800872	Modulation of IL-10 production	[40]
***IL10***	rs1800890	Modulation of IL-10 production	[41]
***TGFB1***	rs6957	Alteration of macrophage balance and fibrosis	[42]
***TGFB1***	rs1800469	Modulation of TGFβ production; prognostic factor in lung cancer patients	[43, 44]
***TGFB1***	rs1800471	Modulation of TGFβ production; association with the clinical presentation of NHL patients	[18, 45]
***TGFBR1***	rs334348	Risk for breast cancer; alteration of specific miRNA binding sites within *TGFBR1* gene	[46]
***TGFBR2***	rs3087465	Alteration of TGFBR2 promoter activity; prognostic factor in thyroid papillary carcinoma	[47, 48]
***IL17A***	rs3748067	Risk for gastric cancer; association with breast cancer molecular subtype and prognosis	[49, 50]
***IL17F***	rs763780	Alteration of IL-17F downstream pathways	[51]

(^*^) References addressing previous associations with cancer susceptibility, progression or with functional alterations of the respective gene/protein. NHL = non-Hodgkin lymphoma; FL = follicular lymphoma; ALL = acute lymphoid leukemia.

### DNA extraction and genotyping

DNA was extracted from the peripheral blood of patients and controls using the lithium chloride technique [52]. The DNA yield (ng/uL) and purity (260/280 and 260/230 ratios) assessments were performed using a spectrophotometer (NanoDrop^®^ 2000, ThermoFisher Scientific). The final concentration of all samples was set to 50 ng/uL.

SNP genotyping was performed on the Taqman^®^ OpenArray^®^ System (which is based on quantitative PCR reactions). Briefly, the DNA samples were pipetted together with Taqman^®^ Openarray^®^ Master Mix on 384-well plates. The mixture was transferred to genotyping plates by the Openarray^®^ Accufill^TM^ system. Thermocycling was performed over 40 cycles, and the visualization of the polymorphic alleles was achieved by VIC™ and FAM™ fluorophores. A single reaction allowed for the simultaneous detection of all 16 polymorphic variants.

### Immunohistochemistry

The immunohistochemical reactions were performed using TMA slides to address the TME composition. A broad panel of TME markers was selected, which encompassed subpopulations of TILs, macrophages and FDCs (CD3, CD4, CD8, CD57, FOXP3, PD1, granzyme B, perforin, CD23, CD68, CD163, iNOS). In addition, for each gene in the SNP approach, the respective protein was detected by immunohistochemistry (IL-12A, IL-2, IL-10, TGFβ, TGFBR1, TGFBR2, IL-17A, IL-17F). Details on the clones and dilutions of each antibody are listed in Supplementary Table 5.

The TMA slides were subjected to antigen retrieval using citric acid/pH 6.0 or EDTA/pH 9.0 buffers. Endogenous peroxidase activity was blocked using a 3% hydrogen peroxide aqueous solution for 20 minutes. The antigen-antibody reaction was performed overnight at 4°C and was then amplified using a third-generation polymer that was tagged with anti-mouse and anti-rabbit immunoglobulins and horseradish peroxidase (HRP; Novolink Polymer Detection System, Leica Biosystems, Newcastle Upon Tyne, UK). The reactions were developed with the 3-3′-diaminobenzidine (DAB) chromogenic substrate (Sigma, D5637, St. Louis, MO, USA). Hence, the positive cell structures (membrane, cytoplasm or nuclei) presented as a brown color. Positive and negative controls were run for each batch. The negative control was obtained by omitting only the primary antibody used for the reaction.

IHC stain quantification was done automatically using the Aperio Scanscope XT device and by averaging both of the TMA core scores. The Positive Pixel Count algorithm was used to grade the pixels as negative (N), low positive (LP), positive (P) and high positive (HP) (Figure 1). The inputs for the algorithm were a hue value of 0.1, a hue width of 0.5 and a color saturation threshold of 0.1 (for most cores). In cases with nonspecific background, the color saturation threshold was increased to 0.15 to minimize false-positive detection. For the antibodies that stained specific TME populations (*e.g.* CD68, FOXP3, CD3), the fraction of all the positive pixels was considered as the score ($\text{Score}=\frac{LP+P+HP}{LP+P+HP+N}$). For the antibodies that heterogeneously stained both the tumor and microenvironment cells (all the cytokine antibodies), we calculated the H-score [53] using the following formula:

$$\text{HSCORE}=3\left(\frac{HP}{LP+P+HP+N}\right)+2\left(\frac{P}{LP+P+HP+N}\right)+1\left(\frac{LP}{LP+P+HP+N}\right)$$

For the TIL subpopulations, we also performed “qualitative analyses” by describing the predominant pattern of proliferation [5, 7, 8]. In this setting, there were 4 possible patterns as follows: “intrafollicular” (TILs homogeneously localized inside neoplastic follicles); “perifollicular” (TILs concentrated in the periphery of neoplastic follicles); “interfollicular” (TILs more visualized in the interfollicular spaces); and “diffuse” (TILs well distributed randomly inside and outside the follicles). For statistical purposes, “intrafollicular” and “perifollicular” were both considered “follicular” patterns, whereas “interfollicular” and “diffuse” were reclassified “non-follicular.”

For the CD23 stains, we used a “pattern” approach. Thus, the FDC categories were as follows: absent (Group 1); a minority of neoplastic follicles with disrupted meshworks (Group 2); the majority of neoplastic follicles with well- developed meshworks (Group 3); and uniformly well-developed meshworks (Group 4) [54].

### Statistical analyses

We performed the chi-squared (χ^2^), Fisher’s exact test, Mann Whitney’s test, and Spearman’s correlation index to assess the bivariate associations. When necessary, numerical variables were dichotomized as “high” and “low,” based on the median levels.

In the SNP study, we tested, for each variant, the HWE, using the χ^2^ goodness-of-fit test. Pairwise LD analyses were performed to estimate the haplotype formation using Haploview 4.2 (https://www.broadinstitute.org/haploview/haploview). LD was measured by the disequilibrium coefficient (D′), and LD significance was considered at a D′ ≥ 80%.

An exploratory PCA with Varimax rotation was used to address the interactions among the IHC scores. The PCA included both the numerical and categorical variables (pixel counting and patterns of tumor-infiltrating lymphocytes, respectively).

Agglomerative, unsupervised hierarchical clustering was also performed using the numerical IHC data (pixel counting) and using Euclidean distances and Ward’s clustering algorithm.

Survival analyses were performed only for the patients that received R-CHOP or R-CVP as a frontline therapy. The OS was defined as the time from diagnosis until death from any cause or the last follow-up. The EFS was defined as the time from diagnosis until death from disease, disease progression or the last follow-up. The survival curves were plotted using the Kaplan-Meier method and were compared using a log-rank test. We further performed Cox univariate regressions for the variables that influenced survival based on the Kaplan-Meier curves. Finally, a Cox multivariate model was proposed, including variables with a *p* value ≤ 0.10 in the univariate analysis. The genotyping and IHC data were evaluated separately in the survival models. The prognostic roles of both the PCA and clusters were also examined.

A final *p* value < 0.05 was considered statistically significant. When necessary, corrections for multiple comparisons were performed using the Benjamini-Hochberg method.

A CONSORT diagram (Supplementary Figure 3) summarizes the workflow of the study, as well as the number of patients for both the IHC and SNP analyses.

## SUPPLEMENTARY MATERIALS




