# Supplementary material for: An integrative microenvironment approach for follicular lymphoma: roles of inflammatory cell subsets and immune-response polymorphisms on disease clinical course
Source: Oncotarget. 2020 Aug 18;11(33):3153–73. doi: 10.18632/oncotarget.27698 (PMC7443366; doi:10.18632/oncotarget.27698)
Supplement: Supplementary file 1 [file oncotarget-11-3153-s001.pdf]

An integrative microenvironment approach for follicular lymphoma: roles of inflammatory cell subsets and immune-response polymorphisms on disease clinical course

SUPPLEMENTARY MATERIALS

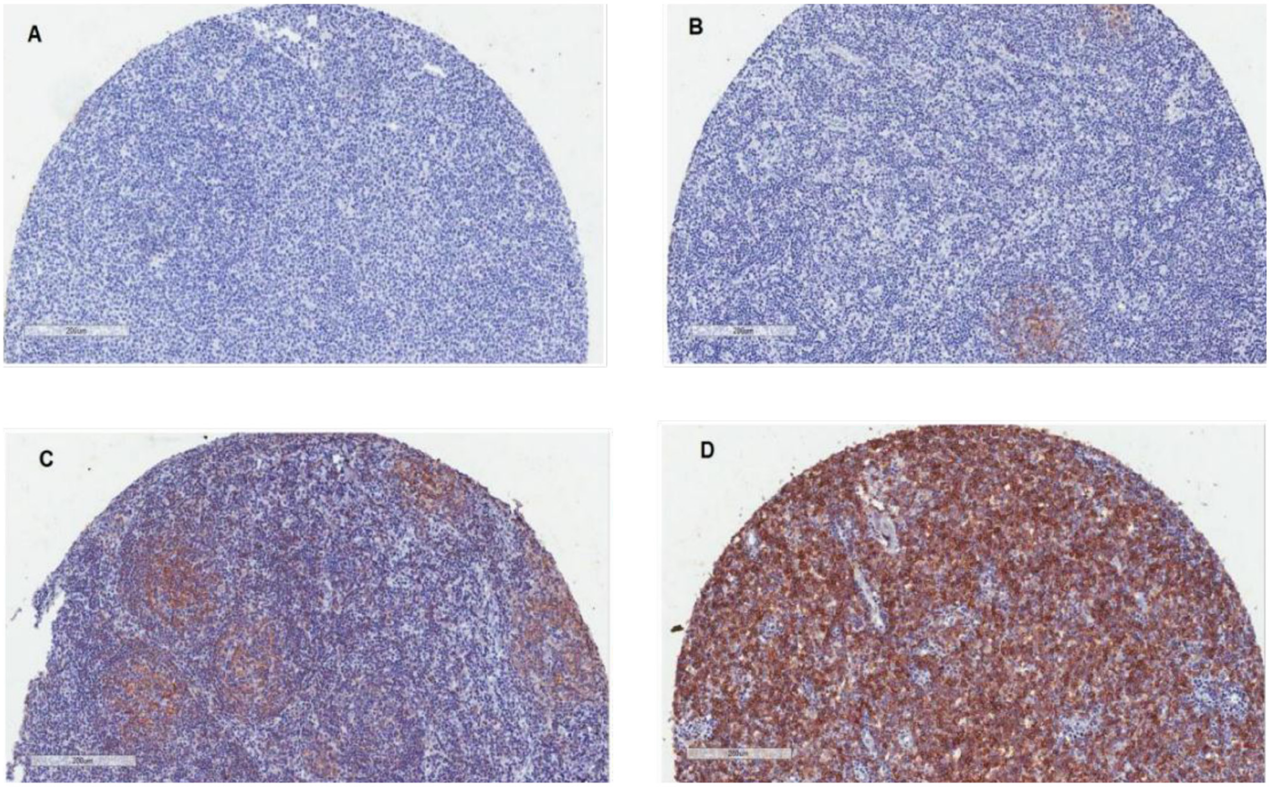

Supplementary Figure 1: Follicular dendritic cells' meshworks classification in follicular lymphoma. (**A**) Group 1: absent meshwork, (**B**) Group 2: minority of the follicles with disrupted meshworks, (**C**) Group 3: majority of the follicles with well-developed meshworks, (**D**) Group 4: uniformly well-developed meshworks.

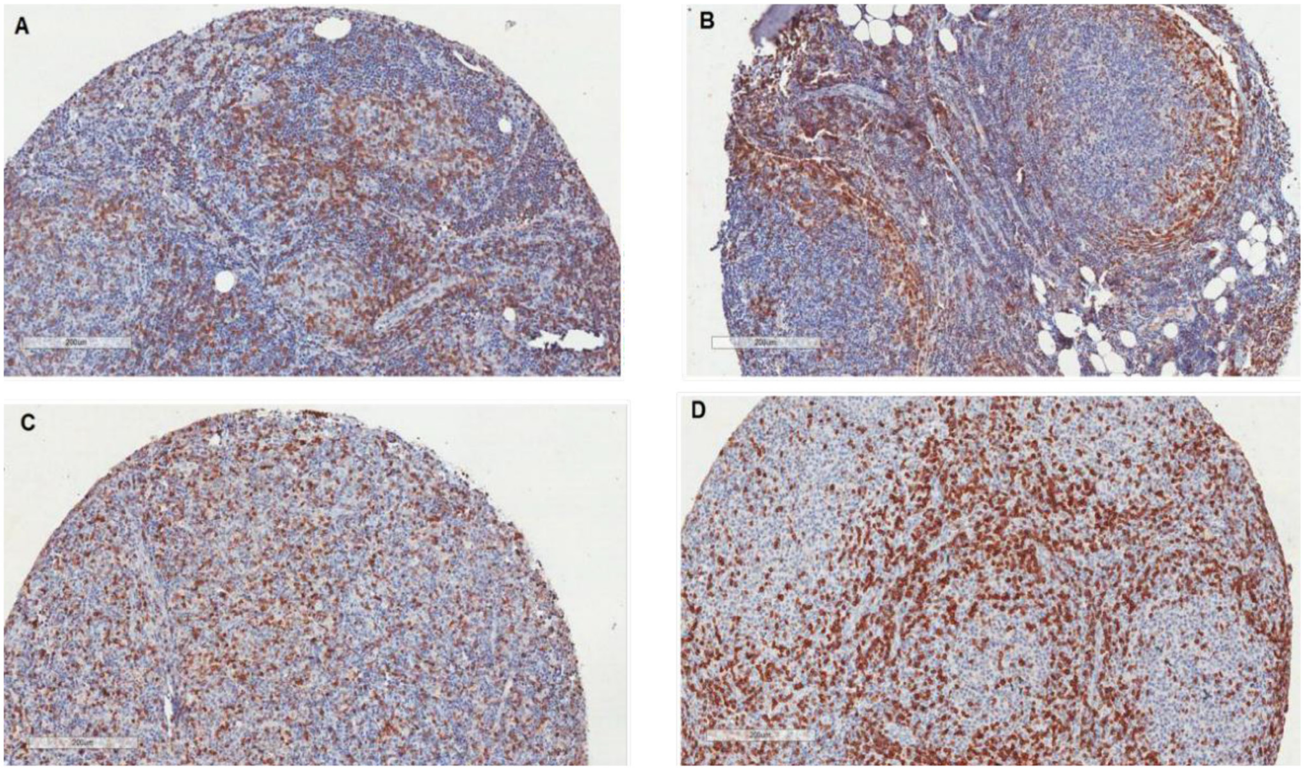

Supplementary Figure 2: Illustration of the predominant patterns of tumor-infiltrating lymphocytes in follicular lymphoma. (**A**) Intrafollicular and (**B**) Perifollicular patterns belong to the "follicular" classification. (**C**) Diffuse and (**D**) Interfollicular patterns belong to the "non-follicular" classification. All images were captured from CD3 stainings.

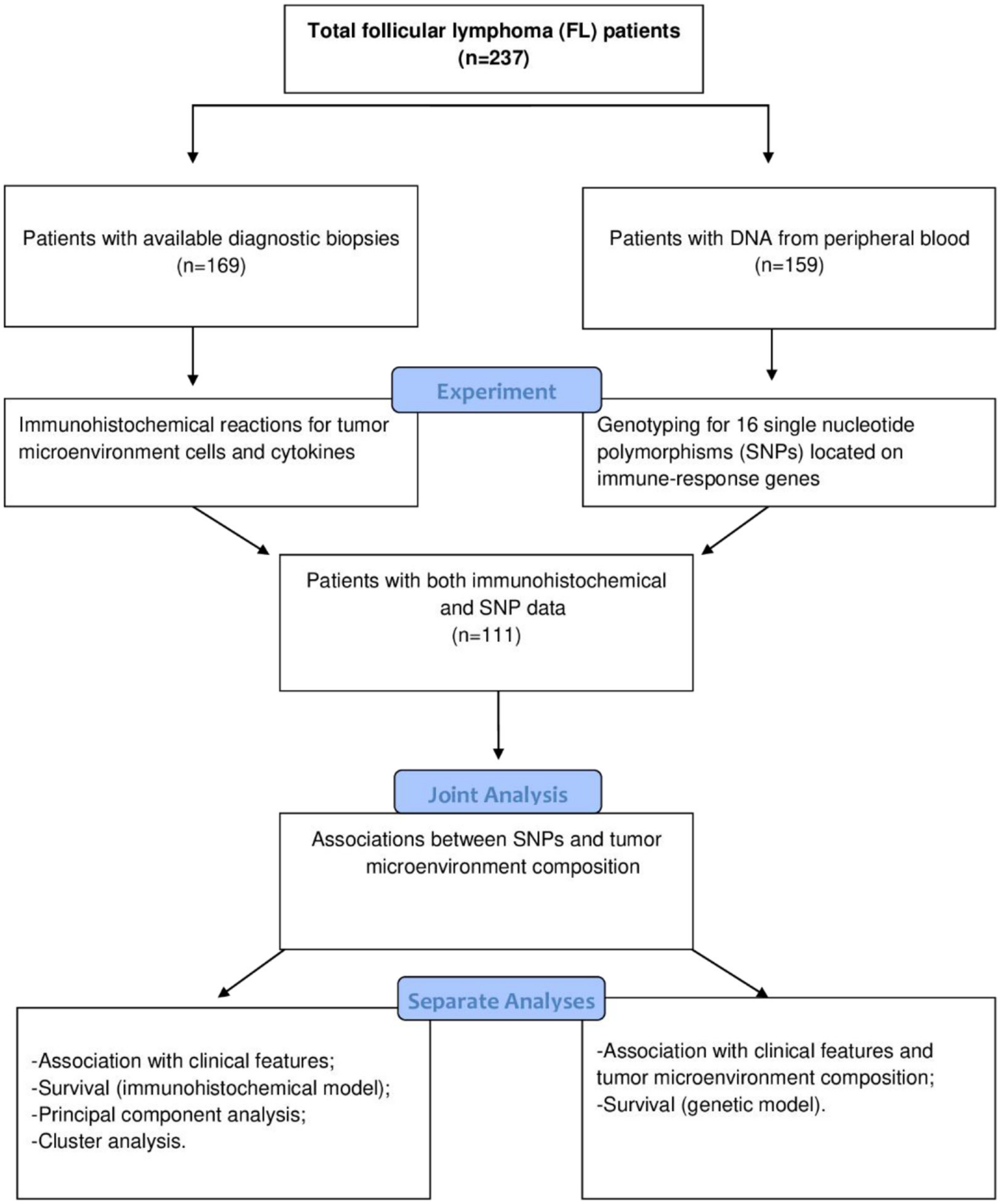

Supplementary Figure 3: CONSORT diagram showing the workflow of experiments and analyses performed in the present study.

Supplementary Table 1: Distribution of the infiltration patterns of T and NK lymphocytes in follicular lymphomas

	Follicular pattern		Non-folicular pattern	
Marker	Intrafollicular	Perifollicular	Interfollicular	Diffuse
**CD3 (%)**	9 (6.4)	6 (4.3)	85 (60.7)	40 (28.6)
**CD4 (%)**	9 (7.2)	7 (5.6)	61 (48.8)	48 (38.4)
**CD8 (%)**	6 (4.5)	7 (5.3)	91 (68.4)	29 (21.8)
**FOXP3 (%)**	13 (10.4)	25 (20.0)	0 (0.0)	87 (69.6)
**PD1 (%)**	116 (77.9)	0 (0.0)	0 (0.0)	33 (22.1)
**CD57 (%)**	56 (44.8)	19 (15.2)	0 (0.0)	50 (40.0)

Supplementary Table 2: T-cell patterns of infiltration in follicular lymphoma and clinicopathological features. See Supplementary Table 2

Supplementary Table 3: Hardy-Weinberg equilibrium calculation for the patients enrolled in the study

SNP (gene)	HWE assessment
rs568408 *(IL12A)*	n = 151; χ^2 = 4.85<br>**p = 0.02**
rs755004 (*IL12A*)	n = 157; χ^2 = 1.75<br>p = 0.18
rs485497 (*IL12A*)	n = 155; χ^2 = 0.17<br>p = 0.67
rs583911 (*IL12A*)	n = 151; χ^2 = 0.07<br>p = 0.77
rs2069762 (*IL2*)	n = 151; χ^2 = 5.45<br>**p = 0.01**
rs6822844 (*IL2*)	n = 157; χ^2 = 2.02<br>p = 0.15
rs1800872 (*IL10*)	n = 154; χ^2 = 0.07<br>p = 0.77
rs3024491 (*IL10*)	n = 156; χ^2 = 6.13<br>**p = 0.01**
rs1800890 (*IL10*)	n = 155; χ^2 = 0.77<br>p = 0.37
rs1800469 (*TGFB1*)	n = 159; χ^2 = 2.08<br>p = 0.14
rs1800471 (*TGFB1*)	n = 158; χ^2 = 1.68<br>p = 0.19
rs6957 (*TGFB1*)	n = 154; χ^2 = 1.91<br>p = 0.16
rs334348 (*TGFBR1*)	n = 146; χ^2 = 8.1<br>**p < 0.01**
rs3087465 (*TGFBR2*)	n = 155; χ^2 = 1.01<br>p = 0.30
rs3748067 (*IL17A*)	n = 148; χ^2 = 2.66<br>p = 0.10
rs763780 (*IL17F*)	n = 152; χ^2 = 1.55<br>p = 0.21

Note: Hardy-Weinberg Equilibrium (HWE) is admitted for $p > 0.05$. Different patient numbers for each polymorphism were due to failed genotyping in some cases.

Supplementary Table 4: Frequencies of haplotypes in *IL10* and *IL12A* among follicular lymphoma patients

Gene and haplotype	Estimated frequency
***IL10* CCT**	38.4%
***IL10* CAT**	33.0%
***IL10* ACA**	18.4%
***IL10* CC**[a]	40.4%
***IL10* CA**[a]	33.0%
***IL10* AC**[a]	26.1%
***IL10* CT**[b]	46.3%
***IL10* AT**[b]	33.0%
***IL10* CA**[b]	20.1%
***IL12A* GGG**	42.2%
***IL12A* GAG**	33.6%
***IL12A* GAA**	14.5%
***IL12A* GGA**	42.2%
***IL12A* AGG**	31.7%
***IL12A* AAG**	15.3%
***IL12A* GG**[c]	43.8%
***IL12A* AG**[c]	41.0%
***IL12A* AA**[c]	15.2%
***IL12A* GA**[d]	51.6%
***IL12A* GG**[d]	33.1%
***IL12A* AG**[d]	15.2%

[a]Haplotype between rs3024491 e rs1800872. [b]Haplotype between rs1800872 e rs1800890. [c]Haplotype between rs583911 e rs568408. [d]Haplotype between rs568408 e rs485497.

Supplementary Table 5: Primary antibodies used for immunohistochemistry

Antibody	Dilution	Supplier	Code
**FOXP3**	1:25	BioSB	BSB6763
**Granzyme B**	1:400	BioSB	BSB5594
**Perforin**	1:10	BioSB	BSB2110
**CD57**	Pure	BioSB	BSB5277
**CD3**	1:100	BioSB	BSB5146
**CD4**	1:50	BioSB	BSB5153
**CD8**	1:500	BioSB	BSB5174
**CD23**	1:50	BioSB	BSB5209
**CD68**	1:500	BioSB	BSB5293
**CD163**	1:100	BioSB	BSB6308
**Ki67**	1:50	BioSB	BSB5713
**IL2**	1:800	Abcam	ab92381
**IL10**	1:150	Abcam	ab34843
**IL12A**	1:10000	Abcam	ab131039
**IL17A**	1:50	Abcam	ab136668
**IL17F**	1:25	Abcam	ab168194
**TGFβ**	1:300	Abcam	ab27969
**TGFBR1**	1:50	Abcam	ab31013
**TGFBR2**	1:25	Abcam	ab61213
**PD1**	1:100	Abcam	ab137132
**iNOS**	1:8000	Abcam	ab129372
