# Supplementary material for: An integrative microenvironment approach for follicular lymphoma: roles of inflammatory cell subsets and immune-response polymorphisms on disease clinical course
Source: Oncotarget. 2020 Aug 18;11(33):3153–73. doi: 10.18632/oncotarget.27698 (PMC7443366; doi:10.18632/oncotarget.27698)
Supplement: Supplementary file 2 [file oncotarget-11-3153-s002.docx]

| Supplementary Table 2: T-cell patterns of infiltration in follicular lymphoma and clinicopathological features | | | | | | | | | | | | | | | | |
| --- | --- | --- | --- | --- | --- | --- | --- | --- | --- | --- | --- | --- | --- | --- | --- | --- |
|  | **CD3** | | | **CD4** | | | **CD8** | | | **FOXP3** | | | **PD1** | | | |
|  | **Follicular (%)** | **Non-folicular (%)** | **p** | **Follicular (%)** | **Non-folicular (%)** | **p** | **Follicular (%)** | **Non-folicular (%)** | **p** | **Follicular (%)** | **Non-folicular (%)** | **p** | **Follicular (%)** | **Non-folicular (%)** | **p** |  |
| B symptoms |  |  |  |  |  |  |  |  |  |  |  |  |  |  |  |  |
| Present | 7  (46.7) | 38  (31.1) | 0.50** | 9  (56.2) | 32  (29.9) | 0.12** | 7  (53.8) | 38  (32.2) | 0.22** | 18  (48.6) | 23  (27.1) | 0.08** | 38  (33.6) | 10  (30.3) | 0.96** |  |
| Absent | 8 (53.3) | 84  (68.9) |  | 7  (43.8) | 75  (70.1) |  | 6  (46.2) | 80  (67.8) |  | 19  (51.4) | 62  (72.9) |  | 75  (66.4) | 23  (69.7) |  |  |
| FLIPI |  |  |  |  |  |  |  |  |  |  |  |  |  |  |  |  |
| High risk | 3  (23.1) | 36  (32.4) | 0.75* | 5  (38.5) | 29  (29.3) | 0.98* | 2  (20.0) | 37  (34.3) | 0.65* | 13  (35.1) | 23  (28.4) | 0.46** | 32  (31.1) | 9  (33.3) | 0.82** |  |
| Intermediate/low risk | 10  (76.9) | 75  (67.6) |  | 8  (61.5) | 70  (70.7) |  | 8  (80.0) | 71  (65.7) |  | 24  (64.9) | 58  (71.6) |  | 71  (68.9) | 18  (66.7) |  |  |
| Extranodal disease |  |  |  |  |  |  |  |  |  |  |  |  |  |  |  |  |
| Present | 6 (46.1) | 39 (34.5) | 0.53** | 5 (33.3) | 34 (34.3) | 0.93** | 3 (27.2) | 38 (34.9) | 0.61** | 11 (30.5) | 32 (40.0) | 0.44** | 32 (30.5) | 12 (40.0) | 0.64** |  |
| Absent | 7 (53.9) | 74 (65.4) |  | 10 (66.6) | 65 (65.7) |  | 8 (72.8) | 71 (65.1) |  | 25 (69.5) | 48 (60.0) |  | 73 (69.5) | 18 (60.0) |  |  |
| Bone marrow infiltration |  |  |  |  |  |  |  |  |  |  |  |  |  |  |  |  |
| Present | 11  (73.3) | 52  (42.3) | 0.08***** | 8  (53.3) | 47  (43.5) | 0.77** | 10  (76.9) | 49  (41.5) | **0.04**** | 24  (64.9) | 39  (45.3) | 0.10** | 57  (50.0) | 13  (39.4) | 1.0** |  |
| Absent | 4  (26.7) | 71  (57.7) |  | 7  (46.7) | 61  (56.5) |  | 3  (23.1) | 69  (58.5) |  | 13  (35.1) | 47  (54.7) |  | 57  (50.0) | 20  (60.6) |  |  |

P values were obtained using Chi-squared tests (*) and Fisher’s exact tests (**). All p-values were adjusted for multiple comparisons (Benjamini-Hochberg method).
